# Propofol improves sleep deprivation‐induced sleep structural and cognitive deficits via upregulating the BMAL1 expression and suppressing microglial M1 polarization

**DOI:** 10.1111/cns.14798

**Published:** 2024-07-17

**Authors:** Huan Liu, Chenyi Yang, Xiaoqing Wang, Baochen Yu, Ying Han, Xinyi Wang, Zixuan Wang, Miao Zhang, Haiyun Wang

**Affiliations:** ^1^ The Third Central Clinical College of Tianjin Medical University Tianjin China; ^2^ Nankai University Affinity the Third Central Hospital Tianjin China; ^3^ Tianjin Key Laboratory of Extracorporeal Life Support for Critical Diseases Tianjin China; ^4^ Artificial Cell Engineering Technology Research Center Tianjin China; ^5^ Tianjin Institute of Hepatobiliary Disease Tianjin China; ^6^ Nankai University Tianjin China

**Keywords:** cognitive impairment, microglia, propofol, sleep, sleep deprivation

## Abstract

**Background:**

Sleep deprivation (SD) is a growing global health problem with many deleterious effects, such as cognitive impairment. Microglia activation‐induced neuroinflammation may be an essential factor in this. Propofol has been shown to clear sleep debt after SD in rats. This study aims to evaluate the effects of propofol‐induced sleep on ameliorating sleep quality impairment and cognitive decline after 48 h SD.

**Methods:**

Almost 8–12‐week‐old rats were placed in the SD system for 48 h of natural sleep or continuous SD. Afterwards, rats received propofol (20 mg·kg^−1^·h^−1^, 6 h) via the tail or slept naturally. The Morris water maze (MWM) and Y‐maze test assessed spatial learning and memory abilities. Rat EEG/EMG monitored sleep. The expression of brain and muscle Arnt‐like protein 1 (BMAL1), brain‐derived neurotrophic factor (BDNF) in the hippocampus and BMAL1 in the hypothalamus were assessed by western blot. Enzyme‐linked immunosorbent assay detected IL‐6, IL‐1β, arginase 1 (Arg1), and IL‐10 levels in the hippocampus. Immunofluorescence was used to determine microglia expression as well as morphological changes.

**Results:**

Compared to the control group, the sleep‐deprived rats showed poor cognitive performance on both the MWM test and the Y‐maze test, accompanied by disturbances in sleep structure, including increased total sleep time, and increased time spent and delta power in non‐rapid eye movement sleep. In addition, SD induces abnormal expression of the circadian rhythm protein BMAL1, activates microglia, and causes neuroinflammation and nerve damage. Propofol reversed these changes and saved sleep and cognitive impairment. Furthermore, propofol treatment significantly reduced hippocampal IL‐1β and IL‐6 levels, increased BDNF, Arg1, and IL‐10 levels, and switched microglia surface markers from the inflammatory M1 type to the anti‐inflammatory M2 type.

**Conclusion:**

Propofol reduces SD‐induced cognitive impairment and circadian rhythm disruption, possibly by lowering neuronal inflammation and switching the microglia phenotype from an M1 to an M2 activated state, thus exerting neuroprotective effects.

## Graphical abstract

Acute sleep deprivation leads to cognitive deficits and disturbances in sleep structure, which are associated with microglia activation. Propofol works by inhibiting microglia, promoting the conversion of M1 to M2, reducing neuroinflammation, improving sleep quality, and reversing the cognitive deficits caused by sleep deprivation.

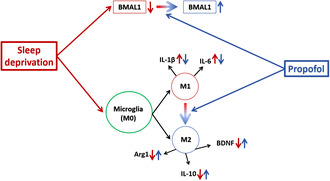

## 1 INTRODUCTION

Sleep is essential for many biological processes,^1^ including learning and memory.^2^ According to the World Health Organization (WHO), the global rate of sleep disorders reaches 27%, and it is estimated that short‐term insomnia affects 30–50% of the population.^3^ Especially in the intensive care unit (ICU), 59–80% of ICU patients experience sleep disruptions such as sleep latency, fragmentation and circadian rhythm disturbances,^4^, ^5^ which are even in ICU patient survivors for at least 12 months.^6^ The physical and psychological effects of sleep deprivation (SD) include cognitive impairment,^7^ anxiety,^8^ depression,^9^ systemic inflammation,^10^ and oxidative stress.^8^ Besides, SD is closely associated with other common ICU complications, such as delirium. Sleep patterns change before the onset of delirium; thus, improving sleep quality in ICU patients can reduce delirium severity.^5^, ^11^

Sleep consists of non‐rapid eye movement (NREM) sleep and rapid eye movement (REM) sleep, and each stage of sleep has unique functions and roles in maintaining the overall cognitive performance of the brain.^12^, ^13^ In particular, NREM sleep may serve as a novel protective cognitive reserve factor in Alzheimer's disease (AD) pathology, and facilitating the quality of slow‐wave sleep in NREM sleep enhances cognitive functioning in older adults and patients with mild cognitive impairment.^14^ Sedative drugs such as benzodiazepines are commonly used to treat sleep disorders in ICU. However, side effects, including cognitive impairment, insomnia rebound after discontinuation, and so on, are of great concern. Moreover, a recent retrospective study showed that benzodiazepine use was not associated with ICU sleep improvement.^5^ Propofol, the most widely used intravenous anesthetic in clinical practice, induces loss of consciousness quickly and reliably and allows rapid and smooth awakening; it has been used in treating patients with refractory chronic primary insomnia and is safe and effective in improving sleep quality long after treatment.^15^

Microglia act as resident immune cells in the central nervous system, secreting various cytokines that are potent molecules involved in sleep and cognition.^16^, ^17^ The activation state of microglia can be divided into a pro‐inflammatory M1 state and an anti‐inflammatory M2 state.^18^ IL‐1β, IL‐6, and TNF‐α are markers of M1 microglia; brain‐derived neurotrophic factor (BDNF), arginase 1 (Arg1) and IL‐10 are markers of M2 microglia.^19^ A growing body of research suggests that SD activates microglia, affects levels of pro‐ and anti‐inflammatory factors, triggers neuroinflammation, impairs neurogenesis, and leads to cognitive impairment.^20^, ^21^ Studies on the non‐anesthetic effects of propofol mainly focus on its anti‐inflammatory and antioxidant effects. Our previous studies confirmed this view: propofol (20 mg·kg^−1^·h^−1^, 6 h) inhibited oxidative stress by regulating the expression of bivalent metal transporter 1.^22^

Although propofol has been reported to lessen the incidence of delirium and postoperative cognitive dysfunction,^23^, ^24^ its effect on SD‐induced cognitive deficits has not been elucidated. Therefore, the present study aimed to investigate the role of propofol in inhibiting microglia hyperactivation, reducing inflammatory levels, and thereby facilitating sleep disorders and cognitive impairment caused by acute SD.

## 2 METHODS

### 2.1 Animals

Male Sprague Dawley rats, weighing 280–350 g, were purchased from Beijing Vital River Laboratory Animal Technology Co., Ltd. The rats were housed in individual cages with free access to food and water, a 12 h light/12 h dark cycle (07: 00–19: 00 light), and temperature control (21°–24°C). Experimental procedures were approved by the Institutional Animal Care and Use Committee, and the Institutional Committee approved all animal experiments for Animal Care and Use of Tianjin Medical University. Every effort was made to minimize animal suffering. The animals were randomly divided into four groups as follows: (1) control group (random sleep–wake, Ctrl); (2) continuous sleep deprivation for 48 h (SD); (3) continuous sleep deprivation for 48 h followed by infusion of propofol 20 mg kg^−1^·h^−1^, 6 h (SD‐P) and (4) continuous sleep deprivation for 48 h followed by natural sleep (SD‐S). The SD‐P group was treated with propofol for 6 h after 48 h SD and then recovered to behavioral tests start the next day. The SD‐S group received SD for 48 h and slept naturally until behavioral studies began the next day.

### 2.2 Sleep deprivation

We chose a SD model established in previous experiments,^25^ using a device purchased from Lafayette Instrument (catalog#Model80391). Briefly, rats in the device were subjected to intermittent tactile stimulation in which a nearly silent metal rod was used to interrupt their sleep. This process avoids any human contact and intervention or introduction of foreign objects, allowing free movement and access to adequate food and water. SD systems placed in noisy environments with prolonged lighting and intermittent sleep disturbances simulate the poor sleep environment of ICU. It is, therefore, superior to other existing methods of sleep interruption. The interruption given by the device was constant and lasted for 48 h (starting at 7:00).

### 2.3 Morris water maze

Morris water maze (MWM) tests were conducted according to the protocol of Wadhwa et al.^26^ with slight modifications. Animals were automatically monitored using a computer‐assisted video tracking system (LabMaze v3.0, Beijing Zhongshi Dichuang Technology Development Co., Ltd). MWM consists of circular pools and platforms that can be moved and hidden beneath the water's surface. It is evenly divided into four quadrants. During the habituation phase, the rats were placed in the pool for 120 s to acclimate to the environment. After that, the rats were put into the pool from four different quadrants every day for four consecutive days, and the platform fixed in a certain quadrant was found within 60 s each time. If no platform was found, the operator guided the rat to the platform and stayed there for 15 s. The screening was conducted 24 h after the last training using a space exploration experiment. If the rats reached the original platform area within 10 s, the rats were well‐trained, and an SD model could be established. After SD and treatment, the SD group entered the no platform test stage on the day after the end of SD, and the SD‐P and SD‐S groups entered the no platform test stage on the day after the end of SD. In this test, rats were put into the pool from the same quadrant and were free to explore for 1 min. Record the escape latency, the number of target platforms crossed, and the time spent in the target quadrant.

### 2.4 Y‐maze test

Y‐maze test can be used to assess short‐term memory in rodents,^27^ which was performed using a horizontal maze consisting of three arms (40 cm long, 30 cm high, and 15 cm wide), each 120 degrees relative to the other. Rats were placed on one arm and allowed to explore the maze freely for 8 min. Make record the animal's movements via video and define arm entry as all four paws entering the arm. Alternating behavior was calculated when the rat entered all three arms consecutively. The percentage of alternation was calculated as (number of alternations)/(total number of arm entries − 2) × 100 (%). After the last experiment of the MWM, the Y‐maze test was performed.

### 2.5 Sample collection

Fresh or perfused brain samples of the hypothalamus and hippocampus were collected under terminal anesthesia at 9 a.m. on the same day after the behavioral tests for western blotting, immunofluorescence, enzyme‐linked immunosorbent assay (ELISA) and other analyses.

### 2.6 Western blot

Rat brain was taken, and the hippocampus and hypothalamus were homogenized in RIPA buffer (Beyotime) containing PMSF and phosphatase inhibitors, and the supernatant was collected by centrifugation at 12,000 rpm for 20 min at 4°C. The protein concentration was measured using the BCA Protein Assay Kit (Beyotime). Electrophoresis was performed on 10% gels, and the separated proteins were transferred to 0.45 μm PVDF membranes, which were blocked with 5% skimmed milk in TBST for 1 h at room temperature and incubated with primary antibodies at 4°C overnight. Primary antibodies included rabbit monoclonal to brain and muscle Arnt‐like protein 1 (BMAL1) (1:1000, Abcam), rabbit monoclonal to BDNF (1:1000, Abcam) and rabbit monoclonal to GAPDH (1:3000, Abcam). Incubate for 1 h at room temperature with secondary antibody (1:5000, Abcam). Blotted proteins were visualized by adding an ECL reagent (Elabscience) and protein expression was quantified using ImageJ (National Institutes of Health).

### 2.7 ELISA

Rat hippocampal tissue was homogenized, then enriched for microglia by centrifugation at 1000 g for 30 min, which were lysed in cell lysis buffer (Solarbio) and centrifuged at 1000 g for 30 min. According to the manufacturer's instructions, use ELISA commercial kits for IL‐6 (Elabscience), IL‐1β (Fine Test), Arg1 (Fine Test) and IL‐10 (Fine Test).

### 2.8 Immunofluorescence

OCT frozen blocks were prepared by cardiac perfusion with saline and 4% paraformaldehyde, followed by the removal of the brain, fixation in the same fixative for 24 overnight, and then dehydration in a 4°C gradient for 24 h in a solution containing 10%, 20%, and 30% sucrose. Sections (20 μm) were subsequently made using a Leica cryostat (Leica CM1800; Leica). Sections were rinsed three times (5 min time^−1^) in 0.01 M PBST (pH 7.3), and then rinsed again after 1 h permeation containing 0.3% TritonX‐100. 5% goat serum closed section 1 h. Image analysis of ionized calcium‐binding adapter molecule 1 (Iba1) staining to quantify microglia regions, and the primary antibody was incubated overnight at 4°C: rabbit anti‐Iba1 (1:500, Abcam). The primary antibody was then washed three times in 0.01 M PBS and then incubated for 2 h at room temperature, protected from light, with the corresponding secondary antibody: AlexaFluor594 coupled goat anti‐rabbit IgG (1:400, Abcam); the wash was repeated three times, DAPI was added for 8 min, and the rinses were performed three times (5 min time^−1^).

As described, the area, number and length of microglial processes in the CA1 region were quantified by skeleton analysis in Image J (National Institutes of Health).^28^ The researchers who analyzed the image were unaware of the animal treatments.

### 2.9 EEG/EMG recording

The rats were anesthetised with isoflurane, and the brain electrodes were then attached to the four cranial screws, which were implanted on the frontal cortex (ML ±1.5 mm, AP: +2.5 mm) and partial cortex (ML ±1.5 mm, AP: −2.0 mm) for EEG recording. EMG electrodes were implanted into the neck muscles. The rats were fed individually for 1 week, and the cables were connected for acclimatization in the last 3 days. The sleep‐deprived rats began the experiment after natural sleep recovery or propofol treatment. The recovery time of rats after stopping propofol injection was similar. After being fully awake, EEG/EMG were performed from 19:00 to 7:00 the next day using the Medusa system (Bio‐Signal Technologies). In this case, there was no difference between the SD group and SD‐S group during sleep monitoring, so it was reduced to three groups. The recorded EEG/EMG data were analyzed using the artificial intelligence‐driven software Lunion Stage, developed by LunionData (https://www.luniondata.com).

### 2.10 Statistical analyses

Statistical analyses were performed using GraphPad Prism software (GraphPad Software). The Shapiro–Wilk test was used to check the normality of the data. Data of normal distribution were expressed as mean ± SD. For multiple group comparisons, one‐way ANOVA was used. Tukey's post‐hoc test was performed for all experiments. All experiments were repeated at least three times, and *p* < 0.05 was considered statistically significant.

## 3 RESULTS

### 3.1 Propofol treatment improves cognitive deficits in sleep‐deprived rats

**FIGURE 1:**
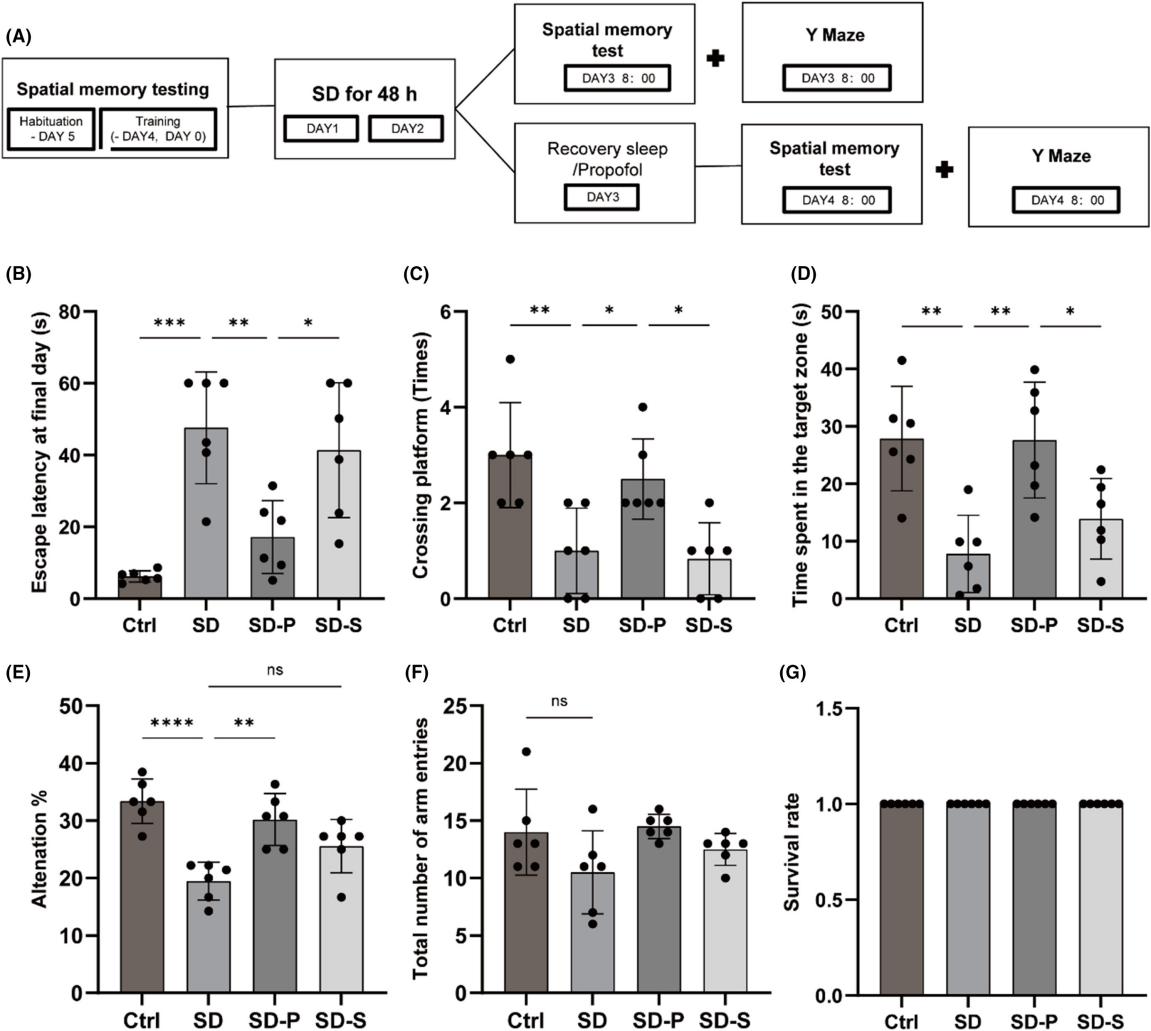
Propofol treatment improves cognitive function in rats impaired by SD. (A) Flowchart of the behavioral tests. MWM test recorded (B) escape latency, (C) number of platform crossing, and (D) risidence time of target quadrant. The Y‐maze recorded (E) percentage of spontaneous alternations, and (F) the total number of arm entries. (G) Survival rate of experimental animals. Data were expressed as mean ± standard deviation, *p < 0.05, **p <0.01, ***p <0.001, ****p<0.0001.

After 48 h of acute SD, the rats developed cognitive dysfunction. To test whether propofol treatment improved cognitive function in sleep‐deprived rats, we performed the MWM test and the Y‐maze test (Figure 1A). In MWM's hidden platform test, sleep‐deprived rats had longer escape latency, fewer crossed platforms times, spent less time in the target quadrant, and did not significantly recover after 24 h of recovery room sleep. However, propofol‐induced sleep significantly improved these defects (Figure 1B–D). We also tested spatial recognition memory in sleep‐deprived rats with Y‐maze test. In the sleep‐deprived rats, the alternation% decreased and increased after propofol treatment, suggesting that of propofol improved memory in SD rats (Figure 1E). There was no statistically significant difference in the total number of arms between the Ctrl group and the SD group, indicating that SD did not affect the motor function of rats (Figure 1F). None of the animals died after the behavioral test (Figure 1G).

### 3.2 Propofol attenuates sleep architecture disturbances after SD

**FIGURE 2:**
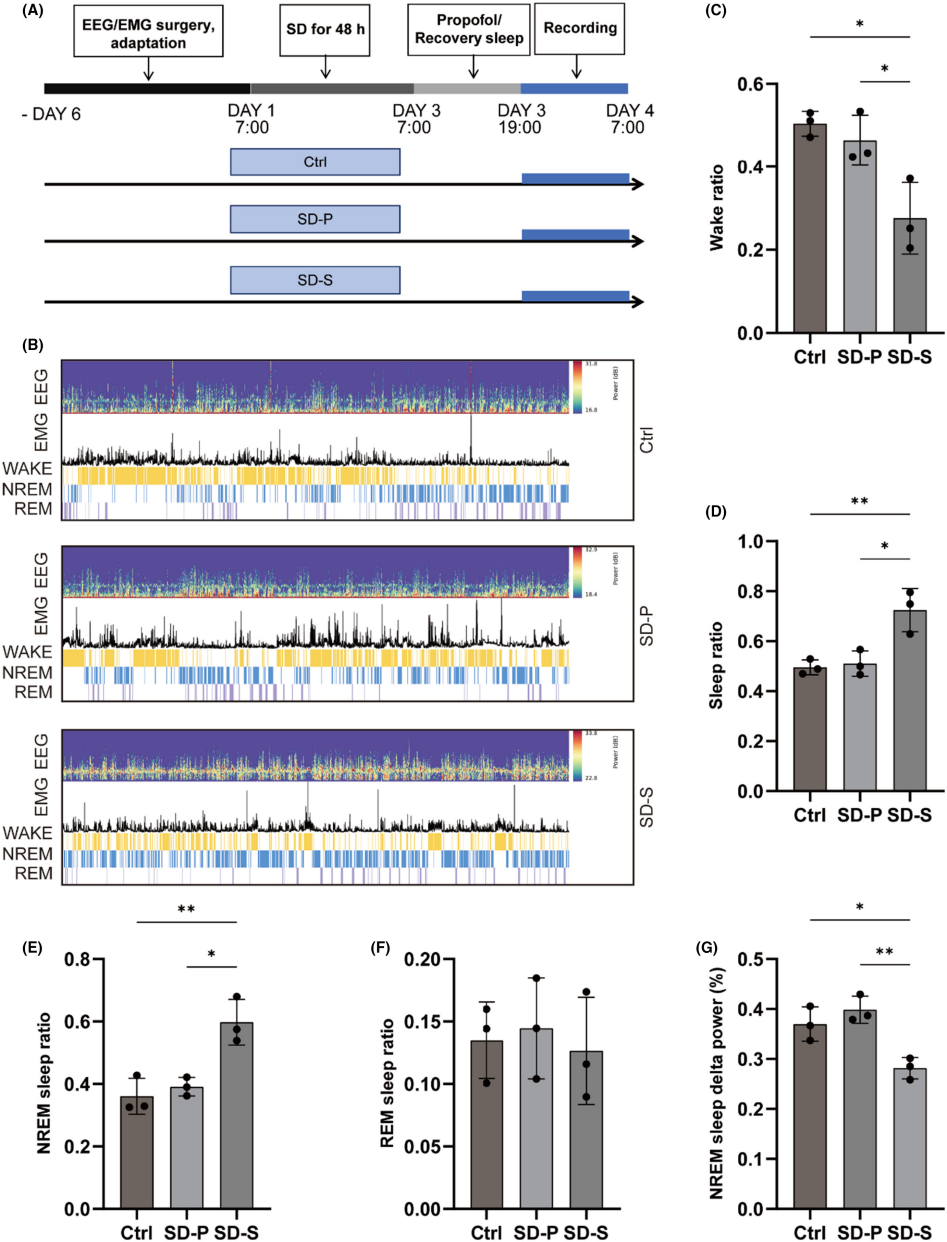
Propofol treatment ameliorates sleep architecture disorders in SD‐impaired rats. (A) Flowchart of EEG/EMG test, recording of (B) sleep architecture, including (C) wake ratio, (D) sleep ratio, (E) NREM sleep ratio, and (F) REM sleep ratio duration. (G) Different treatments produced EEG delta power (0.5–4.0 Hz) of NREM sleep. Data were expressed as mean ± standard deviation, *p < 0.05, **p <0.01.

To further investigate the effect of 48 h SD on subsequent sleep and the protective effect of propofol, an EEG/EMG‐based analysis of alertness status was performed 12 h after SD (Figure 2A). 48 h SD caused significant disruption of sleep architecture (Figure 2B). Compared with Ctrl group, the SD group experienced a substantial reduction in wakefulness and an increase in total sleep duration after 12 h of natural sleep (Figure 2C,D), with altered NREM sleep occupancy ratios (Figure 2E). In contrast, propofol treatment restored SD‐induced sleep structural alterations, but the change in the duration of REM sleep staging was not statistically significant (Figure 2F). Finally, the changes in distributions of delta (0.5–4.0 Hz) percentages in NREM sleep were analyzed (Figure 2G). Increased delta power in EEG spectra during NREM sleep in propofol‐treated sleep‐deprived rats.

### 3.3 Propofol regulates the abnormal clock factor expression and nerve damage

**FIGURE 3:**
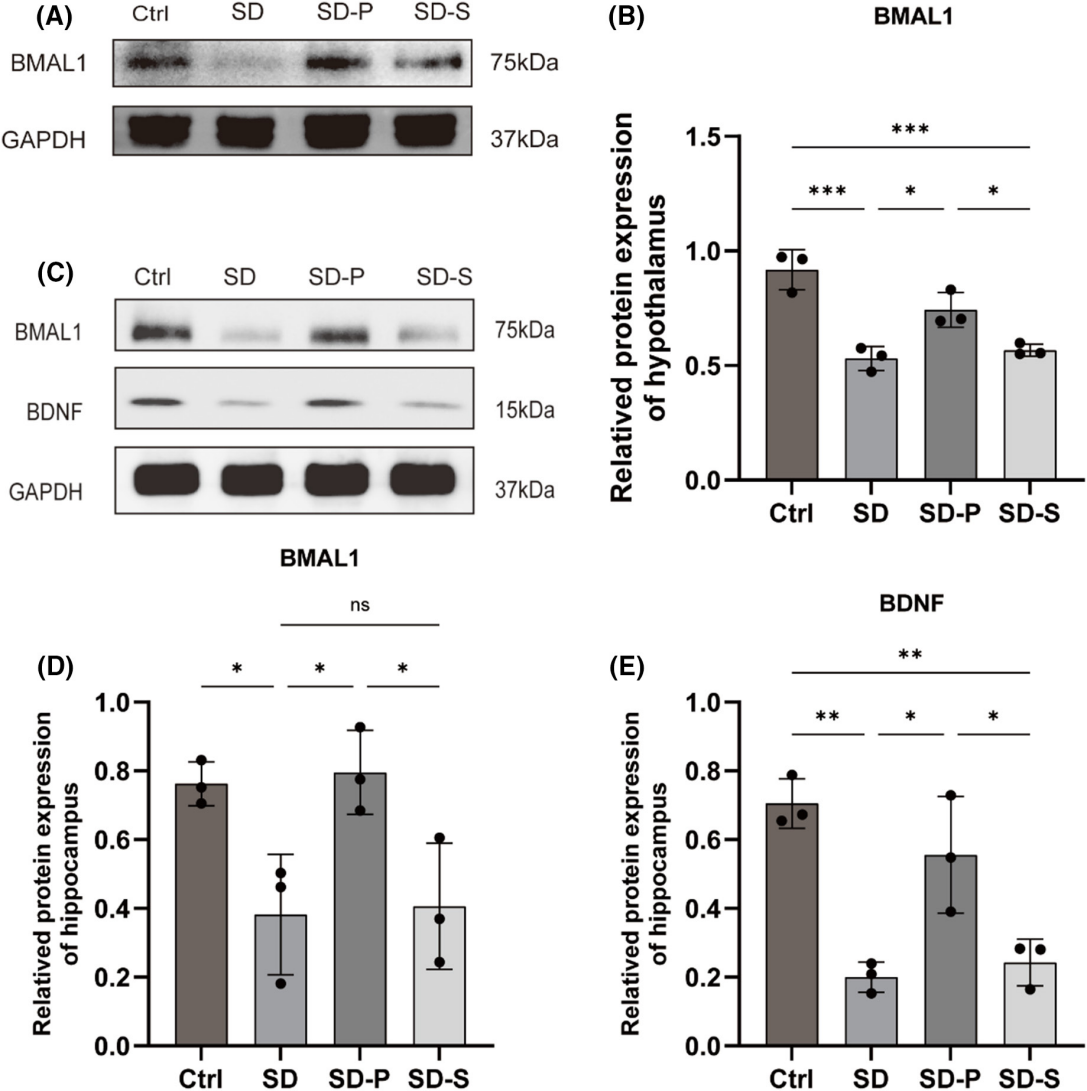
Propofol regulates clock factor expression abnormality and nerve damage. (A, B) Hypothalamic BMAL1 protein expression protein blots and semi‐quantification, (C–E) hippocampal tissue BMAL1 and BDNF expression protein blots and semi‐quantification. Data were expressed as mean ± standard deviation, *p < 0.05, **p <0.01, ***p <0.001.

To verify whether the core clock factor *bmal1* plays a role in the regulation of circadian rhythms and cognitive functions by propofol, Western Blot was used to detect the expression of BMAL1 in the hypothalamus (Figure 3A,B), and the expression of BMAL1 and BDNF in hippocampal tissue (Figure 3C–E). The results showed that the expression of positive regulators BMAL1 and BDNF was suppressed in the SD group and could not fully recover after natural sleep. The expression of BMAL1 and BDNF was elevated in the SD‐P group compared with the Ctrl group. Propofol‐induced sleep can improve the abnormal expression of master clock factors and alleviate the neurological injury effects.

### 3.4 Propofol inhibits hippocampal neuroinflammation in sleep‐deprived rats and promotes microglia shift to an anti‐inflammatory phenotype

**FIGURE 4:**
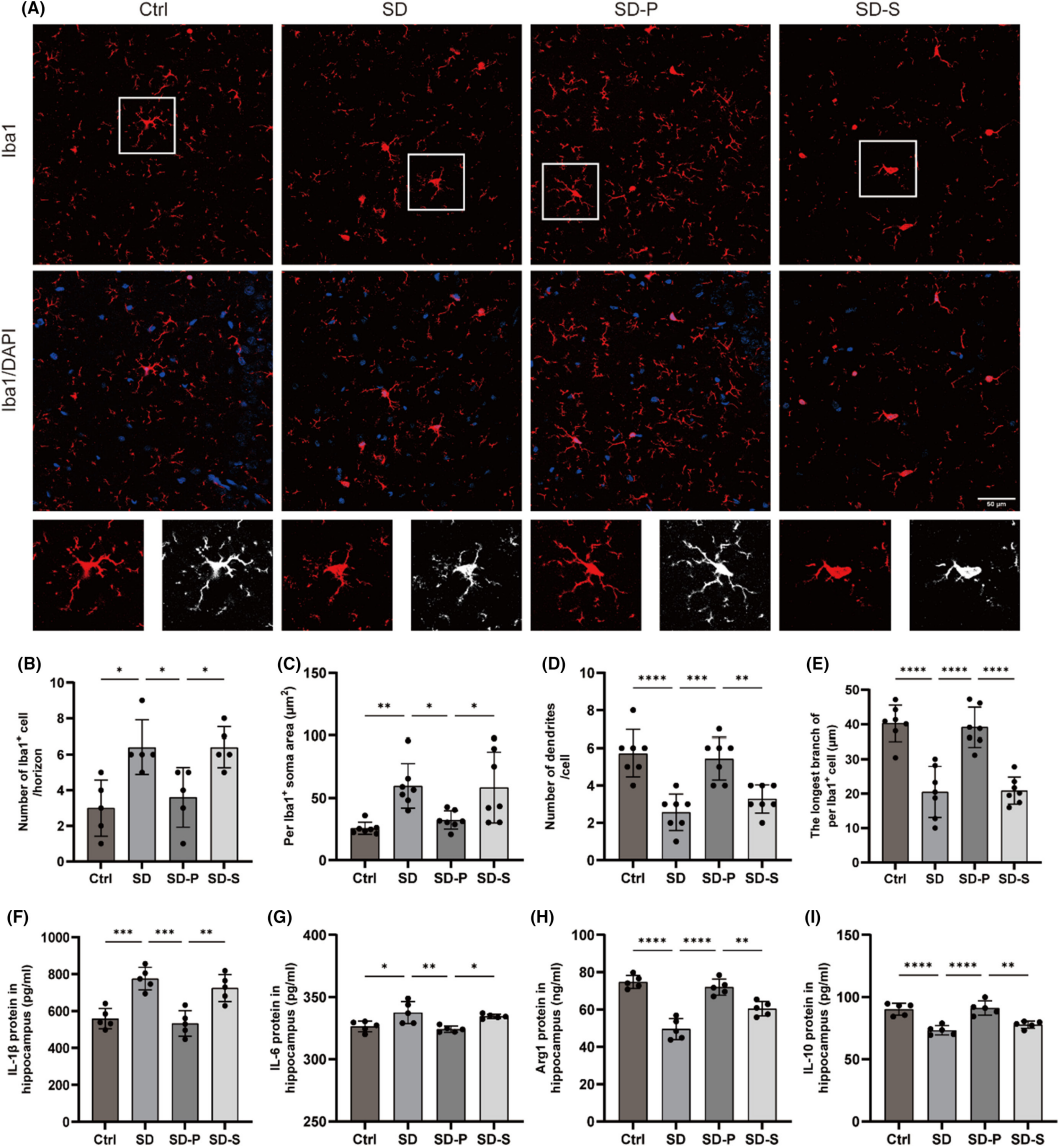
Propofol inhibits hippocampal microglia activation in sleep‐deprived rats. (A–E) Immunofluorescence morphological analysis of microglia, Scale bar: 50 μm. (F, G) Levels of M1‐type microglia surface markers (IL‐1β, IL‐6) were detected, (H, I) and levels of M2‐type microglia surface markers (Arg1, IL‐10) were detected. Data were expressed as mean ± standard deviation, *p < 0.05, **p <0.01, ***p <0.001, ****p <0.0001.

Evidence shows that neuroinflammation in the hippocampus is directly related to cognitive impairment, including microglia activation and increased inflammatory mediators.^29^ We first examined microglia activation in the hippocampus using Iba1 immunostaining (Figure 4A). Acute SD caused a significant increase in the total number of Iba1 cells in the hippocampus (Figure 4B). We further analyzed microglia morphologically by measuring the microglia area, number of branches and length of the longest branch in the hippocampal region (Figure 4C–E). Microglia in the hippocampal of sleep‐deprived rats exhibited activated shapes with larger soma, fewer branches, and shorter lengths. Notably, propofol treatment reduced the number of cells and normalized the morphology of Iba1.

The 48 h SD increased the expression of M1 microglia‐associated factors (IL‐1β and IL‐6) (Figure 4F,G), while decreased the expression of M2 microglia‐associated factors (BDNF, Arg1 and IL‐10) in the hippocampus (Figures 3C and 4H,I). Propofol significantly reversed these effects of acute SD, transforming microglia from a pro‐inflammatory M1 phenotype to an anti‐inflammatory M2 phenotype.

## 4 DISCUSSION

The current study found that SD leads to cognitive deficits, disturbed sleep architecture, and microglia activation. Propofol works by inhibiting microglia, promoting M1–M2 transition, reducing neuroinflammation, improving sleep quality, and ultimately reversing cognitive deficits caused by SD. Propofol shows tremendous therapeutic potential in the treatment of ICU sleep disorders, although this approach requires further research.

Sleep deprivation leads to severe cognitive deficits, including reduced attention and learning,^30^, ^31^ which is consistent with our view. Even more, both clinical and preclinical studies have shown that SD leads to neurodegenerative changes such as AD, which may be related to the worsening of pathological changes in Aβ and Tau.^32^, ^33^ There has been widespread interest in the superior cognitive protective effects of propofol. Propofol and surgery do not affect neuropathological alterations and cognition in 3xTgAD Alzheimer transgenic mice.^34^ What's more, propofol improves postoperative serum levels of Aβ‐42 and Tau proteins and improves postoperative cognitive function in patients with hepatocellular carcinoma compared with inhalation anesthesia.^35^ This may be attributed to the propofol avoiding apoptosis and hyperphosphorylation of Tau proteins through the GSK‐3β pathway,^36^ and in addition, propofol may reduce p‐Tau accumulation by regulating the expression of SOD and HO‐1 via the p62/Keap1/Nrf2 pathway.^37^ And more importantly, ICU patients who were in an unarousable state for ≥24 h due to continuous sedation with propofol or midazolam, compared to midazolam sedation, propofol sedation is associated with a lower risk of subsequent delirium.^38^ Therefore, we concluded that propofol improves cognitive deficits in sleep‐deprived rats, but the underlying mechanism is unclear.

It has been reported that after acute SD, the total sleep duration of rats increases, known as sleep rebound, including NREM sleep and REM sleep duration.^39^ We also observed an increase in sleep duration in sleep‐deprived rats but only an increase in NREM sleep, with no effect on REM sleep. This difference may be related to the sleep model paradigm and EEG/EMG observation time, which deserves further study. Propofol‐induced sleep has many neuro mechanistic overlaps with natural sleep,^40^ with the induced loss of consciousness causing extensive changes in the reorganization of the rich‐clubs in the human brain, ranging from the restructuring of the higher‐order cognitive networks to the reorganization of the rich‐clubs of the primary sensory cortex and cerebellar networks; propofol promotes the normalization of increased release of disordered glutamate and γ‐aminobutyric acid in the hippocampus of paradoxical‐sleeping rats,^41^, ^42^ both of which are similar to those of natural sleep. More importantly, long‐term sedation with propofol in rats does not lead to SD.^43^ When the sleep‐deprived rats were treated with propofol, there was almost no sleep rebound, the rate of recovery to the control level was faster than that of the natural sleep group, and the NREM sleep delta power increased, indicating that part of the sleep debt was compensated during propofol anesthesia and the sleep quality was better than that of natural sleep. This is different from other anesthetics, such as volatile anesthetics (isoflurane and sevoflurane), and the REM sleep rebound after exposure to volatile anesthetics is in sharp contrast to the effect of propofol.^44^

Quality sleep is highly dependent on the expression of circadian rhythms with specific proteins regulated by the supraoptic nucleus of the hypothalamus.^45^ The transcription‐translation negative feedback cycle composed of clock genes such as *Bmal1* maintains the circadian rhythm of the 24‐h cycle of animal cells. *Bmal1* is crucial and independent in clock genes relative to other clock genes, and the loss of *bmal1* disrupts the robust oscillation of the core clock components.^46^ Meanwhile, *bmal1* is not only critical in circadian rhythms but is also closely linked to cognition.^47^, ^48^ Lack of *bmal1* has been shown to lead to learning and memory deficits, and *bmal1* knockout mice exhibit memory loss and a reduced ability to form new short‐term memories,^49^ consistent with our observations. In addition, chronic SD decreases the BMAL1 expression in AD mice, exacerbating the excessive abnormal phosphorylation of Tau and accelerating the development of AD pathology.^50^ Thus, acute SD reduces hippocampal BMAL1 levels in rats, leading to hippocampal‐dependent spatial learning and impaired working memory. Propofol treatment attenuates acute SD‐induced cognitive impairment and sleep structural disturbance by upregulating BMAL1 expression.

There is growing evidence that microglia are implicated in sleep regulation, are involved in sleep–wake state transitions, and that sleep dysfunction due to abnormal microglia activity is prevalent in related disorders such as AD and parasitosis.^51^, ^52^ This also suggests that microglia could be a potential target for improving sleep quality. SD activates a peripheral immune response that triggers inflammatory storms.^53^ Acute SD exacerbates inflammation throughout the body by disrupting circadian rhythms.^10^ The lack of the *bmal1* exacerbates the inflammatory response.^54^, ^55^ Under the action of multiple factors, SD activates microglia and increases the levels of inflammatory cytokines IL‐1β and IL‐6, which induces neuroinflammation. Moreover, microglia activation was evidenced by increased reactivity and deviation from normal microglia morphology. As macrophages in the central nervous system, microglia have a highly plastic morphology closely related to their biological state. We observed that in the resting state, microglia were highly branched; after SD, microglia were rapidly activated, with larger cytosol and shorter dendrites. The cell number of activated microglia increased. Meanwhile, propofol sleep treatment reduced activated microglial counts after SD. In contrast, sevoflurane and isoflurane induce neurotoxicity in neonatal mice through microglial activation, neuroinflammation, and neurotoxicity.^56^, ^57^

Our study also confirmed that the BDNF expression was reduced by SD and increased by propofol treatment, suggesting that the neuroprotective effects of propofol may be related to the release of BDNF. BDNF has been reported to have a variety of neurobiological roles, including protection of neuronal dendritic spine morphology and synaptic plasticity,^58^, ^59^ and underlies cognitive processes in the hippocampus.^60^ BDNF can also exert anti‐inflammatory effects by directly acting on microglia.^61^ Therefore, propofol may inhibit microglial activation via BDNF. Coincidentally, M2‐type microglia can also secrete BDNF,^62^ and promote learning‐related synapse formation and control neuronal excitability through BDNF signaling,^62^, ^63^ which play essential physiological functions in learning and memory. This implies that propofol may promote the transformation of microglia into M2 phenotype and thus play an anti‐inflammatory role. Surprisingly, we further observed a decrease in the expression of M2‐associated markers by acute SD, implying that SD activates microglia towards the M1 phenotype and inhibits M2 activation. Propofol sleep normalized the expression of inflammatory factors in the hippocampus of sleep‐deprived rats, thereby improving homeostasis in the hippocampal environment and facilitating learning and memory. It is worth mentioning that sleep is a very complex and unknown process, closely related to inflammatory factors,^64^ and the rebalancing of inflammatory factors is also a rebalancing of sleep homeostasis and reaffirms the role of propofol in improving sleep architecture.

In conclusion, studies have shown that propofol‐induced sleep, at least to some extent, contributes to the transformation of microglia phenotype and the rebalancing of inflammatory factor production, improving acute SD‐induced neuroinflammation, neurologic damage, sleep structural disorders, and cognitive impairment, indicating the importance of propofol in this recovery. Therefore, our findings support propofol‐induced sedation and sleep homeostasis, which may be beneficial for sleep disorders in ICU patients. Propofol may be a good choice for surgical anesthesia in patients with sleep disorders.

## AUTHOR CONTRIBUTIONS

Study concept and design: HW, HL, and CY. Experimental studies: HL and CY. Acquisition, analysis, or interpretation of data: HL and CY. Drafting of the manuscript: HL and CY. Critical revision of the manuscript for important intellectual content: All authors. Statistical analysis: HL, CY. Obtained funding: HW and CY. Administrative, technical, or material support: XW, BY, YH, XW, ZW, and MZ. Study supervision: HW.

## CONFLICT OF INTEREST STATEMENT

The authors declare that the research was conducted independent of any commercial or financial relationships that could be construed as a potential conflict of interest.

## Data Availability

The data that support the findings of this study are available from the corresponding author upon reasonable request.
